# Immune checkpoint inhibitor-associated gastritis complicated by cytomegalovirus infection: A rare case report

**DOI:** 10.1055/a-2646-1624

**Published:** 2025-08-01

**Authors:** Pingfan Mo, Wenli Xu, Yanqin Long

**Affiliations:** 1 71069 Department of Gastroenterology, The First Affiliated Hospital of Zhejiang University School of Medicine, Hangzhou, China

We report the case of a 60-year-old Chinese woman admitted to the hospital presenting with persistent vomiting and abdominal pain for one month. She had a history of endometrial cancer treated with surgery two years ago followed by serplulimab (a PD-1 inhibitor) therapy. Initial gastroscopy at a local clinic showed no abnormalities, which prompted a referral to our institution. Upon admission, a diagnostic gastroscopy revealed extensive gastric exfoliative changes without involvement of the esophagus or duodenum, and a nasojejunal tube was placed for nutritional support ( Video 1 , Fig. 1 ). Serological and pathological testing further confirmed a cytomegalovirus infection in the gastric mucosa ( Fig. 2 ). Treatment was therefore initiated with methylprednisolone (80 mg intravenous injection daily) and ganciclovir (0.5 g orally three times daily) with clinical improvement.

Gastroscopy showing gastric mucosal changes in the patient with PD-1 inhibitor-associated gastritis complicated by cytomegalovirus infection.Video 1

**Fig. 1:**
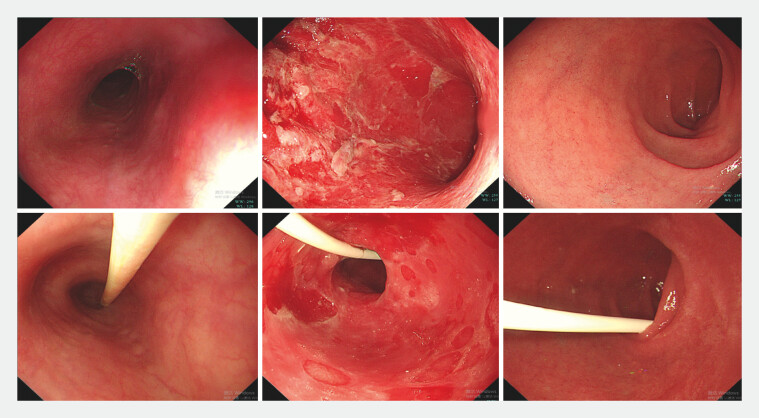
Image during gastroscopy showing signs of extensive gastric mucosa damage without involvement of the esophagus or duodenum in the patient with PD-1 inhibitor treatment and cytomegalovirus infection. A nasojejunal tube was placed for nutritional support.

**Fig. 2:**
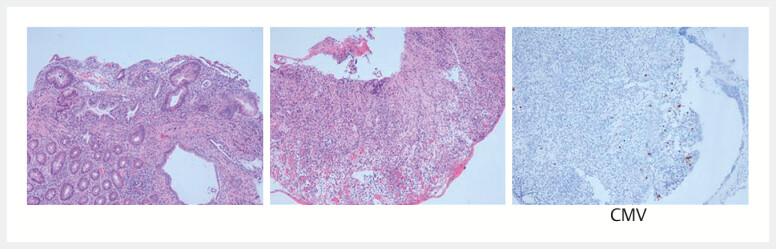
Histologic appearance showing small fragments of granulation tissue with inflammatory necrotic exudate, consistent with ulceration, accompanied by cytomegalovirus infection (magnification × 40).

Since immune checkpoint inhibitors have significantly transformed the therapeutic landscape of malignancies 1 , immune-related adverse events have emerged as a concomitant challenge that can involve multiple organ systems 2 . This case describes a rare manifestation of a PD-1 inhibitor-related gastritis complicated by a cytomegalovirus infection, which was characterized by isolated gastric mucosal damage in the gastrointestinal tract. The combination of steroid and antiviral therapy proved to be effective in managing this condition. Interestingly, although the interval between the gastroscopy performed at our hospital and the one conducted at the local clinic was less than one month, the results differed significantly, even though the patient had undergone immune checkpoint inhibitor therapy for nearly two years. In addition, we provide detailed imaging and video documentation to support recognition by endoscopists and clinicians.

Endoscopy_UCTN_Code_CCL_1AB_2AC_3AZ

## Notes

Endoscopy E-Videos https://eref.thieme.de/e-videos
*E-Videos* is an open access online section of the journal *Endoscopy* , reporting on interesting cases and new techniques in gastroenterological endoscopy. All papers include a high-quality video and are published with a Creative Commons CC-BY license. Endoscopy E-Videos qualify for HINARI discounts and waivers and eligibility is automatically checked during the submission process. We grant 100% waivers to articles whose corresponding authors are based in Group A countries and 50% waivers to those who are based in Group B countries as classified by Research4Life (see: https://www.research4life.org/access/eligibility/ ). This section has its own submission website at https://mc.manuscriptcentral.com/e-videos .

## References

[1] Drew MP The blockade of immune checkpoints in cancer immunotherapy Nat Rev Cancer 2012 12 252 264 22437870 10.1038/nrc3239 PMC4856023

[2] Ying J Jingwen Y Douglas BJ Harnessing big data to characterize immune-related adverse events Nat Rev Clin Oncol 2022 19 269 280 35039679 10.1038/s41571-021-00597-8

